# Causality in Statistical Power: Isomorphic Properties of Measurement, Research Design, Effect Size, and Sample Size

**DOI:** 10.1155/2016/8920418

**Published:** 2016-03-17

**Authors:** R. Eric Heidel

**Affiliations:** Department of Surgery, Office of Medical Education, Research, and Development, University of Tennessee Graduate School of Medicine, 1924 Alcoa Highway, Knoxville, TN 37920, USA

## Abstract

Statistical power is the ability to detect a significant effect, given that the effect actually exists in a population. Like most statistical concepts, statistical power tends to induce cognitive dissonance in hepatology researchers. However, planning for statistical power by an* a priori* sample size calculation is of paramount importance when designing a research study. There are five specific empirical components that make up an* a priori* sample size calculation: the scale of measurement of the outcome, the research design, the magnitude of the effect size, the variance of the effect size, and the sample size. A framework grounded in the phenomenon of isomorphism, or interdependencies amongst different constructs with similar forms, will be presented to understand the isomorphic effects of decisions made on each of the five aforementioned components of statistical power.

## 1. Introduction

Statistical power, like statistics in general, tends to induce feelings of cognitive dissonance in people [1]. Statistical power is the ability to detect treatment effects, given that they truly exist in the population [2]. In more general terms, statistical power is the chance that researchers will achieve a significant *p* value. In the applied sense, hepatology researchers must balance several different empirical factors that have causal and isomorphic effects on statistical power.

Isomorphism is the phenomenon where constructs that are different in content, but similar in form, are linked due to their interdependent associations [3]. The constructs of measurement, research design, magnitude and variance of effect size, and sample size are all isomorphic in their effects on statistical power and each other. A change in one construct will cause a predictable change in statistical power, as well as the other respective constructs. The etymology of the word isomorphism comes from the Greek “isos,” meaning equal, and “morphe,” meaning form [4]. In essence, the constructs of measurement, research design, magnitude and variance of effect size, and sample size are equal in their causal effects on the formation of statistical power in applied research.

A biostatistician at a regional medical center in southeastern United States conducted several thousand consultations with residents, fellows, faculty, physicians, and staff over the course of eight years.* A priori* sample size calculations continually proved to be the hardest part of assisting novice and expert researchers in the planning stages of conducting research. Consultees oftentimes had no idea what type of effect size should be detected in their respective studies. The biostatistician created a conceptual framework grounded in isomorphism, or the interdependencies that exist amongst different constructs, to better understand how the choices made by consultees had causal effects on statistical power. These isomorphic relationships are presented in Table 1.

## 2. Methods

In order to conduct an* a priori* sample size calculation to achieve adequate statistical power, hepatology researchers must make decisions about (1) the scale of measurement of the outcome, (2) the research design, (3) the magnitude of the effect size, (4) the variance of the effect size, and (5) the sample size that can feasibly be collected [2]. Researchers further have to understand how their choices will ultimately affect statistical power. Decisions made by researchers related to these five constructs will have causal effects on statistical power. The underlying isomorphic reasoning associated with making decisions related to the five empirical components when conducting an* a priori* sample size calculation was presented. The strengths and weaknesses of decisions made by researchers in regard to each of the five components were identified.

## 3. Results

### 3.1. Statistical Power and Measurement of Outcomes

Measurement plays a central role in the ability to detect significant treatment effects [2]. Precision and accuracy in measurement leads to more statistical power. Continuous level measurement of outcomes (interval, ratio, and count) leads to more statistical power and smaller sample sizes. More powerful parametric statistics are used with continuous outcomes. There is inherent measurement error and lack of precision in ordinal and categorical outcomes, leading to decreased statistical power and larger sample sizes required to detect significant effects. Less powerful nonparametric statistics are used with these types of outcomes.

### 3.2. Statistical Power and Research Designs

Research designs are used to answer research questions [5]. However, within-subjects designs provide much more statistical power and significantly smaller sample sizes in applied research. The increase in statistical power comes from participants serving as their own controls. Between-subjects designs decrease statistical power and require larger sample sizes to detect significant effects between independent groups. Multivariate designs will always decrease statistical power and necessitate larger sample sizes to be able to detect confounding effects [6].

### 3.3. Statistical Power and Magnitude of Effect Size

Planning for the effect size is perhaps the hardest part of planning a research study [2]. First, researchers have to specify the scale of measurement of the outcome to know if they are detecting differences in proportions or means and standard deviations. Then, depending upon the research design being used, researchers hypothesize their effect sizes between independent groups, within subjects, or in a multivariate fashion.

The best choice, by far, when planning an* a priori* sample size calculation is to use the means, standard deviations, proportions, and odds ratios presented in the empirical literature. This is called using an* evidence-based measure of effect size* in applied research. Researchers can increase the internal validity of their studies by using effect sizes reported in the literature [5]. Using an* evidence-based measure of effect size* also demonstrates more empirical rigor on their part to plan for a valid effect size and achieve adequate statistical power.

There are two components of an effect size that have isomorphic relationships with statistical power: the magnitude of the effect size and the variance of the effect size [5]. The magnitude of an effect size is the relative difference or change in an outcome expected as a result of treatment or group membership. The variance of an effect size is defined as the amount of homogeneity (or heterogeneity) that is expected in an outcome as a result of treatment.

Large effect sizes will increase statistical power and decrease the needed sample size. Larger effect sizes are easier to detect and require fewer observations if the hypothesized effect holds true in the population. Small effect sizes will always decrease statistical power and increase the needed sample size. In order to detect the nuances of variance within a small effect size, significantly more observations of the outcome will be needed to obtain adequate statistical power.

### 3.4. Statistical Power and Variance of Effect Size

Homogeneity or limited variance in an outcome will increase statistical power and decrease the needed sample size. Group differences or changes in outcomes will be more easily detected in groups of participants that are similar. Inversely, heterogeneity or extensive variance in an outcome reduces the ability to detect significant effects due to such wide dispersion of outcomes in groups or across time.

In applied statistical practice, researchers should overestimate the variance of a given effect size. Overestimating the variance of an effect forces researchers to collect larger sample sizes. While this is not always feasible, the benefits of the overestimation are twofold. Statistical power increases as the sample size grows larger and the precision and accuracy of the treatment effect is greatly improved. In theory, more of the diversity and variance in a given population is represented with larger sample sizes, leading to more generalizable statistical findings [7].

### 3.5. Statistical Power and Sample Size

With larger sample sizes, the chances of detecting significant effects increase drastically. Larger sample sizes allow researchers to detect both small and large effect sizes, regardless of their respective variances. Large sample sizes allow for* flexibility* in detecting treatment effects. Large sample sizes increase flexibility by being able to detect a wide spectrum of effect sizes (small, large, homogeneous, and heterogeneous). Small sample sizes significantly decrease statistical power and decrease the flexibility of detecting any type of effect size. With larger sample sizes, validation techniques such as bootstrapping, split-group, and jack-knife methods can be used to increase precision and accuracy of statistical findings [8].

## 4. Discussion

Statistical power is critical when conducting hepatology research. In order to bolster the understanding of this nebulous and cognitive dissonance-inducing construct in hepatology research, isomorphism can be applied as a framework to make better decisions when designing observational and experimental research. Causality in statistical power is associated with the interdependent and isomorphic relationships between measurement, research design, effect size, and sample size. The decisions made by researchers in regard to the aforementioned constructs will have causal effects on statistical power.

The decisions made by hepatology researchers are completely dependent upon the research question being asked and the current empirical environment. Sometimes, researchers will have to make decisions that they know will decrease statistical power. Many outcomes of interest in medicine are measured at a categorical level. Between-subjects designs are important for understanding group differences related to treatment effects. Multivariate designs can account for confounding variables when predicting outcomes. Small effect sizes that exist in heterogeneous populations may push a clinician past a test or treatment threshold. Small sample sizes may be the only feasible choice when researching rare types of outcomes and disease states. However, the most important thing for researchers to remember is that statistical power is influenced in a causal manner by the decisions made when conducting an* a priori* sample size calculation. Isomorphism is a framework that can assist researchers in designing the most powerful studies given the current research question and research environment.

### 4.1. Scoring Methodology for Statistical Power

While writing the paper, the authors designed a scoring rubric containing every combination of scale of measurement, research design, magnitude, variance, and sample size. This rubric can be used to identify combinations of these five isomorphic constructs that lead to feasible and powerful research designs. The scoring methodology has not been validated formally in the literature but presents a pretty straightforward and user-friendly method for “grading” the combinations of decisions made by researchers in the preliminary phases of a study. A total of 72 possible combinations of the five empirical constructs were identified.

In terms of scoring in the instrument, if the chosen scale of measurement of the outcome was categorical or ordinal, a “0” was given. If the outcome was measured at a continuous level, then a “1” is given. This is because increased precision and accuracy in continuous level measurement increases statistical power. For research designs, a value of “0” is given to between-subjects designs and multivariate designs and “1” is given to the within-subjects design because each participant serves as their own control, leading to more statistical power. With magnitude of effect size, “0” is assigned to a small effect size and “1” denotes a large effect size, because large effect sizes are easier to detect, thus increasing power. Limited variance of outcome is coded as “1” because homogeneity leads to clear delineations between independent groups. Heterogeneity is coded as “0” because it is harder to detect effects in highly diverse populations, decreasing statistical power. Finally, a large sample size was coded as “1” because large sample sizes increase statistical power and increase the ability to detect all kinds of effect sizes. Small sample sizes were coded as “0.” All the 72 rows were summed and statistical power values were created. The score ranges from 0 to 5 with increasing values denoting more statistical power as per the decisions made by researchers. The rating scale for 72 different research designs with respective statistical power scores is presented in Table 2.

## 5. Conclusion

The choices that hepatology researchers make related to measurement of outcomes, research design, magnitude and variance of effect size, and sample size have causal effects on statistical power in applied research. Isomorphism can be used as a framework to increase the understanding of the effects of decisions made by researchers on statistical power. Researchers will be able to make more informed decisions related to* a priori* sample size calculations and design more powerful studies.

## Figures and Tables

**Table 1:** Isomorphism and causal effects on statistical power.

Empirical component	Choice	Effect on statistical power
Measurement of outcome	Categorical outcome	Decreased statistical power
	Ordinal outcome	Decreased statistical power
	Continuous outcome	Increased statistical power

Research design	Between-subjects	Decreased statistical power
	Within-subjects	Increased statistical power
	Multivariate	Decreased statistical power

Magnitude of effect size	Small effect size	Decreased statistical power
	Large effect size	Increased statistical power

Variance of effect size	Homogeneity	Increased statistical power
	Heterogeneity	Decreased statistical power

Sample size	Small sample size	Decreased statistical power
	Large sample size	Increased statistical power

**Table 2:** Scoring methodology for statistical power.

Study	Outcome	Research design	Magnitude	Variance	Sample size	Score
1	Categorical	Between-subjects	Small	Heterogeneity	Small	0
2	Categorical	Multivariate	Small	Heterogeneity	Small	0
3	Ordinal	Between-subjects	Small	Heterogeneity	Small	0
4	Ordinal	Multivariate	Small	Heterogeneity	Small	0
5	Categorical	Within-subjects	Small	Heterogeneity	Small	1
6	Categorical	Between-subjects	Small	Heterogeneity	Large	1
7	Categorical	Multivariate	Small	Heterogeneity	Large	1
8	Categorical	Between-subjects	Small	Homogeneity	Small	1
9	Categorical	Multivariate	Small	Homogeneity	Small	1
10	Categorical	Between-subjects	Small	Homogeneity	Large	1
11	Categorical	Multivariate	Small	Homogeneity	Large	1
12	Categorical	Between-subjects	Large	Heterogeneity	Small	1
13	Categorical	Multivariate	Large	Heterogeneity	Small	1
14	Ordinal	Within-subjects	Small	Heterogeneity	Small	1
15	Ordinal	Between-subjects	Small	Heterogeneity	Large	1
16	Ordinal	Multivariate	Small	Heterogeneity	Large	1
17	Ordinal	Between-subjects	Small	Homogeneity	Small	1
18	Ordinal	Multivariate	Small	Homogeneity	Small	1
19	Ordinal	Between-subjects	Small	Homogeneity	Large	1
20	Ordinal	Multivariate	Small	Homogeneity	Large	1
21	Ordinal	Between-subjects	Large	Heterogeneity	Small	1
22	Ordinal	Multivariate	Large	Heterogeneity	Small	1
23	Continuous	Between-subjects	Small	Heterogeneity	Small	1
24	Continuous	Multivariate	Small	Heterogeneity	Small	1
25	Categorical	Within-subjects	Small	Heterogeneity	Large	2
26	Categorical	Within-subjects	Small	Homogeneity	Small	2
27	Categorical	Within-subjects	Small	Homogeneity	Large	2
28	Categorical	Within-subjects	Large	Heterogeneity	Small	2
29	Categorical	Between-subjects	Large	Heterogeneity	Large	2
30	Categorical	Multivariate	Large	Heterogeneity	Large	2
31	Categorical	Between-subjects	Large	Homogeneity	Small	2
32	Categorical	Multivariate	Large	Homogeneity	Small	2
33	Ordinal	Within-subjects	Small	Heterogeneity	Large	2
34	Ordinal	Within-subjects	Small	Homogeneity	Small	2
35	Ordinal	Within-subjects	Small	Homogeneity	Large	2
36	Ordinal	Within-subjects	Large	Heterogeneity	Small	2
37	Ordinal	Between-subjects	Large	Heterogeneity	Large	2
38	Ordinal	Multivariate	Large	Heterogeneity	Large	2
39	Ordinal	Between-subjects	Large	Homogeneity	Small	2
40	Ordinal	Multivariate	Large	Homogeneity	Small	2
41	Continuous	Within-subjects	Small	Heterogeneity	Small	2
42	Continuous	Between-subjects	Small	Heterogeneity	Large	2
43	Continuous	Multivariate	Small	Heterogeneity	Large	2
44	Continuous	Between-subjects	Small	Homogeneity	Small	2
45	Continuous	Multivariate	Small	Homogeneity	Small	2
46	Continuous	Between-subjects	Small	Homogeneity	Large	2
47	Continuous	Multivariate	Small	Homogeneity	Large	2
48	Continuous	Between-subjects	Large	Heterogeneity	Small	2
49	Continuous	Multivariate	Large	Heterogeneity	Small	2
50	Categorical	Within-subjects	Large	Heterogeneity	Large	3
51	Categorical	Within-subjects	Large	Homogeneity	Small	3
52	Categorical	Between-subjects	Large	Homogeneity	Large	3
53	Categorical	Multivariate	Large	Homogeneity	Large	3
54	Ordinal	Within-subjects	Large	Heterogeneity	Large	3
55	Ordinal	Within-subjects	Large	Homogeneity	Small	3
56	Ordinal	Between-subjects	Large	Homogeneity	Large	3
57	Ordinal	Multivariate	Large	Homogeneity	Large	3
58	Continuous	Within-subjects	Small	Heterogeneity	Large	3
59	Continuous	Within-subjects	Small	Homogeneity	Small	3
60	Continuous	Within-subjects	Small	Homogeneity	Large	3
61	Continuous	Within-subjects	Large	Heterogeneity	Small	3
62	Continuous	Between-subjects	Large	Heterogeneity	Large	3
63	Continuous	Multivariate	Large	Heterogeneity	Large	3
64	Continuous	Between-subjects	Large	Homogeneity	Small	3
65	Continuous	Multivariate	Large	Homogeneity	Small	3
66	Categorical	Within-subjects	Large	Homogeneity	Large	4
67	Ordinal	Within-subjects	Large	Homogeneity	Large	4
68	Continuous	Within-subjects	Large	Heterogeneity	Large	4
69	Continuous	Within-subjects	Large	Homogeneity	Small	4
70	Continuous	Between-subjects	Large	Homogeneity	Large	4
71	Continuous	Multivariate	Large	Homogeneity	Large	4
72	Continuous	Within-subjects	Large	Homogeneity	Large	5

*Note.* Higher scores denote more statistical power.
